# Ultrasound-Assisted Extraction of Antioxidants in Misai Kucing (*Orthosiphon stamineus*)

**DOI:** 10.3390/molecules190812640

**Published:** 2014-08-19

**Authors:** Swee Kheng Ho, Chin Ping Tan, Yin Yin Thoo, Faridah Abas, Chun Wai Ho

**Affiliations:** 1 Department of Food Technology, Faculty of Food Science and Technology, Universiti Putra Malaysia, UPM 43400, Serdang, Selangor, Malaysia; E-Mail: hkheng@hotmail.com; 2 School of Science, Monash University Malaysia, Bandar Sunway, Petaling Jaya 46150, Selangor, Malaysia; E-Mail: thoo.yin.yin@monash.edu; 3 Department of Food Science, Faculty of Food Science and Technology, Universiti Putra Malaysia, UPM 43400, Serdang, Selangor, Malaysia; E-Mail: faridah_abas@upm.edu.my; 4 Department of Food Science and Nutrition, Faculty of Applied Sciences, UCSI University, No. 1, Jalan Menara Gading, UCSI Heights, Cheras 56000, Kuala Lumpur, Malaysia; E-Mail: cwho@ucsiuniversity.edu.my

**Keywords:** ultrasound, extraction, antioxidants, Misai Kucing, *Orthosiphon stamineus*

## Abstract

Ultrasound-assisted extraction (UAE) with ethanol was used to extract the compounds responsible for the antioxidant activities of Misai Kucing (*Orthosiphon stamineus*). Response surface methodology (RSM) was used to optimize four independent variables: ethanol concentration (%), amplitude (%), duty cycle (W/s) and extraction time (min). Antioxidant compounds were determined by total phenolic content and total flavonoid content to be 1.4 g gallic acid equivalent/100 g DW and 45 g catechin equivalent/100 g DW, respectively. Antioxidant activities were evaluated using the 2,2'-azinobis-(3-ethylbenzothiazoline-6-sulfonic acid) (ABTS^•^^+^) radical scavenging capacity assay and the 2,2-diphenyl-1-picrylhydrazyl (DPPH•) radical scavenging capacity assay to be 1,961.3 and 2,423.3 µmol Trolox Equivalent Antioxidant Capacity (TEAC)/100 g DW, respectively. Based on the optimal conditions, experimental values were reported to be close to the predicted value by RSM modeling (*p* > 0.05), indicating the suitability of UAE for extracting the antioxidants of Misai Kucing. Rosmarinic acid, kaempferol-rutinoside and sinesetine were identified by high performance liquid chromatography-mass spectrometry.

## 1. Introduction

Malaysia, with its hot, wet climate and rich diversity of flora and fauna in the heart of the rainforest, has attracted the interest of researchers as a source for natural products. In part the interest in these products is related to people’s concerns about the use of synthetic antioxidants such as butylated hydroxyanisole (BHA) and butylated hydroxytoluene (BHT) in food that may cause undesirable effects on human health [1]. 

In Malaysia, *Orthosiphon stamineus* (known as Misai Kucing) is of interest as it is easily accessible and commonly consumed to treat various ailments [2]. Misai Kucing is famous for its flavonoids which are bioactive due to the presence of phenolic compounds in their structures. Twenty phenolic compounds have been isolated from *Orthosiphon stamineus*, including nine lipophilic flavones, two flavonol glycosides and nine caffeic acid derivatives, such as rosmarinic acid and 2,3-dicaffeoyltartaric acid [3]. The above mentioned caffeic acid derivatives appear to be the most abundant polyphenols in aqueous methanol extracts and the polymethoxylated flavones predominate. The polymethoxylated flavones found in this plant (with flavonoid aglycones being the main compounds) are unique, with a methoxy group at C-5, which is a rare structural feature in flavonoids. Several other classes of chemically active constituents are found in *Orthosiphon stamineus*, such as terpenoids (diterpenes and triterpenes) and sterols. These phenolics found in *Orthosiphon stamineus* have been shown to retard lipid oxidation in biological systems by reducing the oxidative stress. *Orthosiphon stamineus* is also commonly used in Southeast Asia for treating eruptive fever, epilepsy, gallstones, hepatitis, rheumatism, hypertension, syphilis and renal calculus. In Malaysia, *Orthosiphon stamineus* is commercially available in tea bags as a health drink to improve general health and to treat kidney and bladder inflammation, gout and diabetes [4].

In the modern industry, eco-friendly techniques such as ultrasound-assisted extraction (UAE) have gained popularity, as ultrasound irradiation (20–100 kHz) is able to offer high reproducibility, shorter extraction times, reduced solvent consumption, lower temperature and lower energy input compared with other extraction methods [5]. During sonication, ultrasound produces cavitation bubbles from ultrasonic waves that permit greater penetration of the extraction solvent into the plant cell walls compared to conventional extraction methods, effectively releasing the intracellular products of the plant [6]. An ultrasound probe was chosen for this study instead of the more commonly used ultrasonic bath because the bath lacks uniformity in the distribution of the ultrasound energy, and the power of the bath declines with time. By contrast, an ultrasound probe is able to focus on a localized sample zone, which guarantees a higher efficiency extraction [7]. Optimization of the extraction factors was performed using response surface methodology (RSM), which enables evaluation of independent variables and of any interactions with dependent variables [8]. It should be mentioned that several authors have reported concerns over the potential of ultrasonic cavitation to produce free hydroxyl radicals that might cause phenolic degradation [9].

The objective of this work was to optimize the ultrasound assisted extraction factors, including ethanol concentration, amplitude, duty cycle and extraction time, to allow for the maximum extraction of phenolics from *Orthosiphon stamineus* using response surface methodology.

## 2. Results and Discussion

### 2.1. Modeling of UAE on Orthosiphon stamineus

To optimize the ultrasound-assisted extraction (UAE) of antioxidants and phenolic compounds from *Orthosiphon stamineus*, a central composite face-centered (CCF) was designed. The experimental values of total phenolic content (TPC), total flavonoid content (TFC) and total antioxidant activities of *Orthosiphon stamineus* extracts under various experimental conditions are presented in Table 1. The multiple regression coefficients of intercept, linear, quadratic and interaction terms in the experimental model were calculated to fit the response function. To judge the adequacy and fitness of the model, the significance of the regression model, coefficient of determination (*R*^2^), adjusted *R*^2^, lack-of-fit and the coefficient of variation (CV) were used. The value of *R*^2^ should be as close as possible to unity [10] and *R*^2^ should be at least 0.80 to ensure a good fit of the model [11]. The adjusted *R*^2^ is a corrected value for *R*^2^ after the elimination of the unnecessary model terms. If many non-significant terms were included in the model, the adjusted *R*^2^ would be considerably smaller than the *R*^2^ [12]. The values for lack-of-fit have to be non-significant, which is *p* > 0.05 [13]. The coefficient of variation (CV) is a measure of the expression of the standard deviation as a percentage of the mean. The smaller the CV value, the better the reproducibility [14]. Thus, a high value for the CV indicates an unsatisfactory response model.

In this study, a good model fit was obtained for TPC, TFC, ABTS^•^^+^ and DPPH• scavenging capacity, with *R*^2^ values of 0.9975, 0.8994, 0.9528 and 0.9859, respectively, which are all quite close to 1. Adjusted *R*^2^ values for TPC, TFC, ABTS^•^^+^ and DPPH• scavenging capacity were 0.9960, 0.8766, 0.9469 and 0.9776, respectively, with maximum value differences of less than 0.1 compared to their *R*^2^ value. The coefficient of variation for TPC, TFC, ABTS^•^^+^ and DPPH• scavenging capacity fell in the acceptable range of 1.98%−19.13%, indicating that the regression model defined the relationships between the independent factors and responses well, with both the independent factors and responses adequately fitting the experimental data.

### 2.2. Effect of Process Variables on Total Phenolic Content (TPC) and Total Flavonoid Content (TFC)

**Figure 1:**
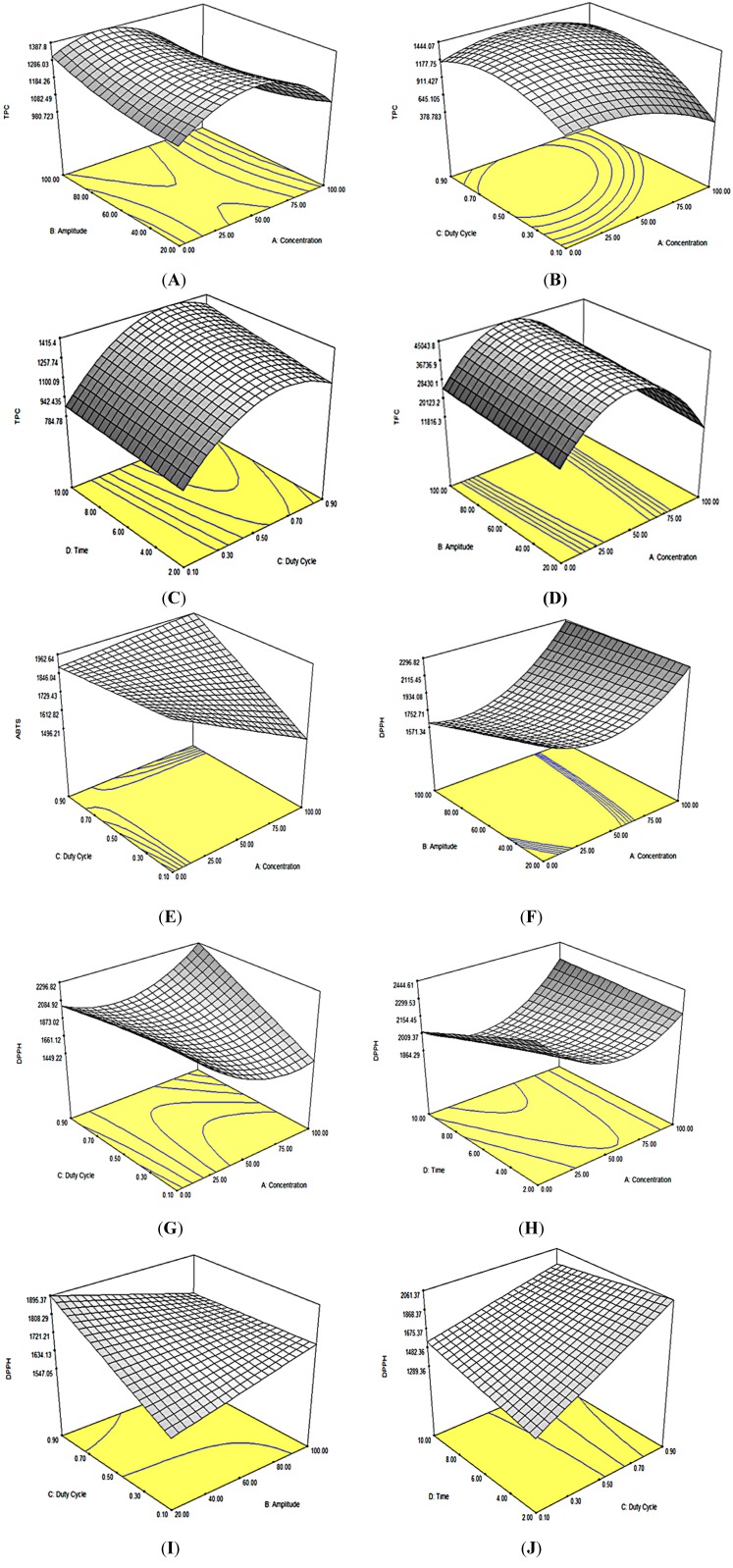
Response surface plots of the total phenolic contents: (**A**) ethanol concentration and amplitude (duty cycle 0.9 W/s and time 10 min); (**B**) ethanol concentration and duty cycle (amplitude 20% and time 10 min); (**C**) duty cycle and time (ethanol concentration 54.77% and amplitude 20%); of the total flavonoid contents: (**D**) ethanol concentration and amplitude (duty cycle 0.9 W/s and time 10 min); of ABTS^•+^ radical cation: (**E**) ethanol concentration and duty cycle (amplitude 20% and time 10 min); of DPPH• radical scavenging activities: (**F**) ethanol concentration and amplitude (duty cycle 0.9 W/s and time 10 min); (**G**) ethanol concentration and duty cycle (amplitude 20% and time 10 min); (**H**) ethanol concentration and time (amplitude 20% and duty cycle 0.9 W/s ); (**I**) amplitude and duty cycle (ethanol concentration 54.77% and time 10 min); (**J**) duty cycle and time (ethanol concentration 54.77% and amplitude 20%), of *Orthosiphon stamineus* extract as affected by ethanol concentration, amplitude, duty cycle and extraction time in UAE.

To determine the optimal levels of the variables for the extraction of phenolic compounds from *Orthosiphon stamineus*, three-dimensional surface plots were constructed (Figure 1A−C) according to Equation (1):

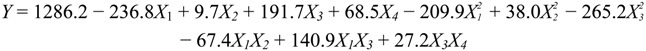
(1)

**Table 1:** Central composite design, experimental data (Expt.) and predicted values (Pred.) of the optimization of ultrasound-assisted extraction (UAE) conditions for the extraction of phenolic antioxidants from *Orthosiphon stamineus*.

Standard Order ^a^	Independent variables								Dependent variables (Responses)															
	*X*_1_^b^		*X*_2_^c^		*X*_3_^d^		*X*_4_^e^		*Y*_1_^f^				*Y*_2_^g^				*Y*_3_^h^				*Y*_4_^i^			
									Expt.		Pred.		Expt.		Pred.		Expt.		Pred.		Expt.		Pred.	
1	0.0		20.0		0.1		10.0		974.0		973.4		13,603.4		14,395.9		1952.3		1959.6		2219.8		2275.4	
2	100.0		20.0		0.1		2.0		265.3		270.3		4696.3		1180.7		1427.8		1497.6		1045.1		1037.8	
3	0.0		100.0		0.1		2.0		1048.9		1045.1		12,134.5		13,983.7		1952.3		1959.6		2216.1		2220.8	
4	100.0		100.0		0.1		10.0		248.3		237.4		8811.1		10,748.8		1571.6		1497.6		1880.2		1845.5	
5	0.0		20.0		0.9		2.0		949.9		937.9		10,730.0		15,920.2		1886.5		1888.2		2438.8		2469.7	
6	100.0		20.0		0.9		10.0		1056.5		1072.4		11,541.1		12,685.3		1957.0		1964.0		2284.9		2321.9	
7	0.0		100.0		0.9		10.0		1284.7		1283.5		38,108.8		25,488.3		1886.5		1888.2		1641.6		1645.1	
8	100.0		100.0		0.9		2.0		726.1		765.5		10,116.7		12,273.1		1959.4		1964.0		2277.5		2263.6	
9	50.0		60.0		0.5		6.0		1283.0		1260.2		36,170.9		37,703.9		1840.0		1827.3		1745.6		1727.5	
10	50.0		60.0		0.5		6.0		1269.2		1260.2		36,170.9		37,703.9		1840.0		1827.3		1785.3		1727.5	
11	0.0		20.0		0.1		2.0		1008.0		992.7		12,728.0		10,402.1		1950.8		1950.8		2219.2		2199.3	
12	100.0		20.0		0.1		10.0		469.0		454.7		6377.8		7167.2		1456.7		1488.8		1393.5		1433.6	
13	0.0		100.0		0.1		10.0		1216.8		1229.5		14,488.6		19,970.2		1950.8		1950.8		2175.6		2158.9	
14	100.0		100.0		0.1		2.0		247.1		256.7		5903.1		6755.1		1455.2		1488.8		1248.2		1311.7	
15	0.0		20.0		0.9		10.0		1206.3		1231.1		17,970.3		21,906.7		1877.7		1879.5		2021.5		1983.9	
16	100.0		20.0		0.9		2.0		996.3		982.8		11,382.8		8691.5		1954.2		1955.3		2214.8		2212.0	
17	0.0		100.0		0.9		2.0		1197.1		1193.9		19,236.4		21,494.5		1918.9		1879.5		2006.9		1992.9	
18	100.0		100.0		0.9		10.0		1096.2		1058.8		17,911.0		18,259.6		1955.3		1955.3		2235.2		2235.6	
19	50.0		60.0		0.5		6.0		1350.7		1362.1		39,810.1		38,700.3		1833.3		1818.6		1665.0		1658.5	
20	50.0		60.0		0.5		6.0		1336.5		1362.1		46,239.4		38,700.3		1833.3		1818.6		1665.0		1658.5	
21	0.0		60.0		0.5		6.0		1264.4		1263.1		18,405.5		13,844.0		1896.6		1928.6		2153.1		2146.6	
22	100.0		60.0		0.5		6.0		783.1		789.6		6640.4		5619.0		1797.1		1735.5		1943.4		1861.0	
23	50.0		20.0		0.5		6.0		1254.6		1264.7		30,314.5		31,811.9		1807.3		1832.0		1823.5		1737.6	
24	50.0		100.0		0.5		6.0		1288.9		1284.0		33,835.8		36,389.9		1807.3		1832.0		1688.1		1705.2	
25	50.0		60.0		0.1		6.0		761.6		779.4		34,785.4		30,843.7		1793.4		1733.2		1586.1		1556.3	
26	50.0		60.0		0.9		6.0		1175.4		1162.7		35,161.2		37,358.2		1893.2		1930.8		1834.5		1886.5	
27	50.0		60.0		0.5		2.0		1175.0		1167.8		31,125.6		31,605.9		1786.5		1832.0		1691.2		1709.4	
28	50.0		60.0		0.5		10		1294.6		1304.8		34,152.3		36,596		1887.5		1832.0		1721.8		1733.4	
29	50.0		60.0		0.5		6		1245.5		1236.3		34,112.8		34,100.9		1825.6		1832.0		1657.3		1721.4	
30	50.0		60.0		0.5		6		1245.5		1236.3		33,736.9		34,100.9		1825.6		1832.0		1679.7		1721.4	

Notes: ^a^ Nonrandomized; ^b^ *X*_1_: Ethanol concentration (%, v/v); ^c^ *X*_2_: Amplitude (%); ^d^ *X*_3_: Duty Cycle (W/s); ^e^ *X*_3_: Extraction time (min); ^f^ *Y*_1_: Total phenolic content (TPC) (mg GAE/100 g dry weight, DW); ^g^ *Y*_2_: Total flavonoid content (TFC) (mg CE/ 100 g dry weight, DW); ^h^ *Y*_3_: 2,2'-azinobis-(3-ethylbenzothiazoline-6-sulfonic acid) (ABTS) radical scavenging capacity (µmol TEAC/ 100 g dry weight, DW); ^i^ *Y*_4_: 2,2-diphenyl-1-picrylhydrazyl (DPPH) radical scavenging capacity (µmol TEAC/ 100 g dry weight, DW).

Figure 1A shows the effects of ethanol concentration and amplitude on the content of total phenolic compounds. As the ethanol concentration increased, TPC increased slowly, then decreased and gradually achieved a saddle shape with an optimum value at approximately 20% ethanol concentration and 60% amplitude. Table 2 shows that ethanol concentration showed substantially more importance than amplitude due to the importance of polarity as reflected by its negative coefficient (*β*_11_ = −209.9). This result shows that the addition of water into the bi-solvent system would influence the polarity, which at 20% yielded more phenolic content compared to 100% according to the “like dissolves like” principle [15]. The effects of concentration and duty cycle are shown in the Figure 1B.

**Table 2:** Regression coefficients, *R^2^*, adjusted *R^2^*, lack of fit test and probability values for the reduced second-order polynomial models of four dependent variables of ultrasound-assisted *Orthosiphon stamineus* extract.

Independent Variables	Regression Coefficient			
	Dependent Variables			
	TPC	TFC	ABTS Radical Scavenging	DPPH Radical Scavenging
	(mg GAE/100 g dry weight, DW)	(mg CE/100 g dry weight, DW)	(µmol TEAC/100 g dry weight, DW)	(µmol TEAC/100 g dry weight, DW)
Intercept, *X*_0_	1286.2	36,835	1826	1702.5
Linear				
*X*_1_, Ethanol concentration	−236.8 ***	−4112.5 ***	−96.6 ***	−142.8 ***
*X*_2_, Amplitude	9.7	2289 *	-	−16.2
*X*_3_, Duty Cycle	191.67 ***	3257.2 **	98.8 ***	165.1 ***
*X*_4_, Extraction time	68.5 ***	2495.1 *	-	12
Quadratic				
*X*_1_^2^	−209.9 ***	−24,369.4 ***	-	282.5 ***
*X*_2_^2^	38.01 **	-	-	-
*X*_3_^2^	−265.2 ***	-	-	-
*X*_4_^2^	-	-	-	-
Interaction				
*X*_12_	−67.4 ***	-	-	97.6 ***
*X*_13_	140.9 ***	-	134.4 ***	260.4 ***
*X*_14_	-	-	-	114.4 ***
*X*_23_	-	-	-	−90.1 ***
*X*_24_	-	-	-	-
*X*_34_	27.2 ***	-	-	−106 ***
Model				
F value	675.6	39.4	161.6	118.9
*p* value	<0.0001	<0.0001	<0.0001	<0.0001
Lack of fit				
F value	7.8	2.9	-	8.7
*p* value	0.058	0.2096	-	0.0504
Mean	1024	22,213.4	1826	1871.9
Standard deviation	20.8	4249	36.1	50.3
R^2^	0.9975	0.8994	0.9528	0.9859
Adjusted R^2^	0.996	0.8766	0.9469	0.9776
Coefficient of variation	2	19.1	2	2.7

Notes: * Significant at 0.05 level; ** Significant at 0.01 level; *** Significant at 0.001 level.

Like Figure 1A, ethanol concentration increased steadily and then decreased. As the duty cycle increased to approximately 0.70 W/s, it was observed that TPC increased, indicating that the proper use of pulse mode can result in increased extraction efficiency and reduced electrical energy consumption. However, the effect of duty cycle on extraction efficiency has been reported as inconsistent. A duty cycle of 50% increased the extraction efficiency of the natural dye from beetroot compared with continuous ultrasound [16], whereas the duty cycle was a significant factor in the ultrasound extraction of phenolic compounds from strawberries [15]. Thus, the effect of the duty cycle on extraction efficiency needs further study. The relationship between duty cycle and extraction time is shown in Figure 1C. Longer extraction time was associated with higher TPC. Similar to Figure 1B, duty cycle increased and then decreased. The maximum duty cycle may not be favorable because the pulse mode of the UAE delivers better extraction efficiency due to non-steady mass transfer of the plant matrix compared with the continuous mode [5].

To determine the optimal levels of the variables for the extraction of flavonoid compounds from *Orthosiphon stamineus*, three-dimensional surface plots were constructed (Figure 1D) according to Equation (2):

*Y* = 36,835 − 4112.5*X*_1_ + 2289*X_2_* + 3257.2*X_3_* + 2495.1*X_4_* − 24,369.4 *X_12_* (2)

All of the variables showed appreciable linear effects, and ethanol concentration showed quadratic effects on TFC. However, no significant interaction effect between any variables was observed. As shown in Figure 1D, at a fixed duty cycle of 0.90 W/s and 10 min, TFC increased steadily up to approximately 50% ethanol concentration, whereas amplitude increased up to 100% yielded the highest TFC observed. A higher amplitude of the sonication probe means a higher intensity of the sonication is transmitted to the plant extract, which increases the sonication effects. However, in some cases, higher amplitude that creates a greater number of cavitation bubbles might dampen the passage of sound energy through the liquid [17].

In this study, we discovered that total flavonoid content (TFC) was significantly higher than total phenolic content (TPC). As mentioned in the introduction, UAE may cause chemical effects due to the free radical production within the cavitation bubbles, resulting in the formation of highly reactive hydroxyl radicals that can combine to form hydrogen peroxide [18].

A free radical is any species that contains one or more unpaired electrons. Common examples of free radicals are oxygen radicals (including superoxide radicals), hydroxyl radicals, and peroxyl radicals. There are also species that are more active than the ground state oxygen molecule which are called reactive oxygen species, such as H_2_O_2_ (hydrogen peroxide), ^1^O_2_ (singlet oxygen), •O^2−^ (superoxide anion) and ^•^OH (hydroxyl radical); these species can damage cells and initiate oxidation of polyunsaturated fatty acids in biological membranes.

Thus, the free radicals produced by UAE might decrease the total phenols in the extract because phenolic compounds will be reduced by the free radicals instead of by the Folin-Ciocalteu reagent (FCR) used in the TPC analysis. In TPC analysis, a reduction of FCR occurs when a phenolic proton dissociates to a phenolate anion. Phenolate and FCR then form a blue compound from the yellow compound produced under alkaline conditions. The darkness of the blue compound indicates the antioxidant concentration. Therefore, the degree of color change is correlated with the sample’s antioxidant concentration [19].

### 2.3. Effects of Process Variables on Antioxidant Activity, ABTS^•+^ Radical Cations and DPPH• Radical Scavenging Capacity

The relationship between the antioxidant activities of *Orthosiphon stamineus* extract and the main variables according to Equation (3) are shown in Figure 1E and according to Equation (4) in Figure 1F–J; three-dimensional surface plots were constructed from these equations:

*Y* = 1826 − 96.6*X*_1_ + 98.8*X_3_* + 134.4 *X_1_X_3_* (3)

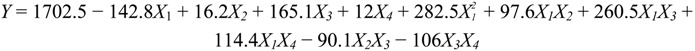
(4)
where Equations (3) and (4) represent the ABTS^•+^ radical cation and DPPH• radical scavenging activities of *Orthosiphon stamineus* extracts, respectively.

The effects of ethanol concentration and duty cycle on ABTS^•^^+^ radical cation activity are shown in Figure 1E. As the ethanol concentration increased, the ABTS^•^^+^ radical cation activity increased gradually and gave the highest activity at 100% ethanol concentration and 0.9 W/s. An interaction effect among the other variables was not observed. However, for DPPH• radical scavenging activity, an interaction effect among all variables except for *X*_2_*X*_4_ was observed (Figure 1F–J). Figure 1F shows the effect of ethanol concentration and amplitude on DPPH• radical scavenging activity. It was observed that a lower ethanol concentration with lower amplitude produced a higher activity. As shown in Figure 1G, high antioxidant activity was observed at constant amplitude and time, at a high ethanol concentration (100%) and high duty cycle (0.9 W/s). At a constant amplitude and duty cycle, it was observed that a low ethanol concentration and short time showed a positive effect on the antioxidant activity of *Orthosiphon stamineus* (Figure 1H)*.* Figure 1F–H reflects a similar trend in their three-dimensional surface plots. However, Figure 1I,J show opposite trends, where a low amplitude (20%) and high duty cycle (0.9 W/s) (Figure 1I) and a short time (2 min) and high duty cycle (0.9 W/s) (Figure 1J) exhibited high antioxidant activity. As mentioned in the previous section, even if the TPC is lower than the value of TFC, it does not affect the antioxidant activity as the high antioxidant activity came mainly from the flavonoids.

### 2.4. Optimal Conditions

Verification experiments were carried out under the optimal conditions to validate the adequacy and suitability of the model equations for predicting the optimum response value. Table 3 shows that the experimental results were very similar to the predicted values, as the *p* values were >0.05 for TPC, TFC, ABTS^˖+^ and DPPH• scavenging capacity. Thus, this result indicates that the response surface modeling could be employed effectively to predict the concentrations of phenolic compounds, ABTS^˖+^ and DPPH• scavenging capacity for *Orthosiphon stamineus* extract.

**Table 3:** Optimum conditions, predicted and experimental values of responses on ultrasound-assisted *Orthosiphon stamineus* extract.

Dependent Responses	Independent Variables				Optimum Value		
	Ethanol Concentration (%, v/v)	Amplitude (%)	Duty Cycle (W/s)	Extraction Time (min)	Experimental ^a^	Predicted	*p*-value
Total phenolic content (TPC) (mg GAE/100 g DW)	15.2	58.5	0.7	8.3	1383.8	1373.7	0.134
Total flavonoid content (TFC) (mg CE/100 g DW)	45.9	100	0.9	10	45,029	45,003	0.938
ABTS radical scavenging capacity (µmol TEAC/100 g DW)	100	99.2	0.9	10	1961.3	1949.6	0.097
DPPH radical scavenging capacity (µmol TEAC/100 g DW)	0	20	0.9	2.4	2423.3	2375.1	0.055
Combination of TPC, TFC, ABTS &DPPH	54.1	20	0.9	10	1332.9	1334.4	0.882
					39793	39,701.3	0.804
					1927.9	1942.2	0.099
					1892.9	1915	0.459

Note: ^a^ Mean of six determinations (*n* = 6) from two replications.

### 2.5. Identification of Phenolics in Orthosiphon stamineus Extract by HPLC-MS

An acetonitrile/water mobile phase system was used for the chromatographic separation. It has been reported that formic acid or phosphoric acid perform well as mobile phase modifiers, as they promote good separation of flavonoids and give a sharp shape to peaks by eliminating peak tailing [20]*.* In this study, the identification of phenolic compounds was carried out by comparing HPLC retention time, the UV spectra and the *m/z* of their molecular ions. Peak 1 was tentatively identified as rosmarinic acid [20] because it had a [*M-*H]^−^ at *m/z* 359 and exhibited characteristic MS^2^ fragments at *m*/*z* 197 [*M-*H-caffeic acid]^−^, 179 [caffeic acid-H]^−^ and 161 [*M-*H-197]^−^, due to the loss of the caffeoyl moiety. Peak 2 was tentatively identified as sinesetine because it exhibited [*M-*H]^+^ at *m/z* 373 [21]. Peak 3 was tentatively identified as kaempferol–rutinoside [22]. The [*M-*H]^+^ at *m/z* 593 produced a major MS^2^ ion at *m/z* 285 (kaempferol) with the loss of *m/z* 308 corresponding to the cleavage of rhamnose-hexose sugar. Thus, one phenolic acid and two flavonoid compounds were identified in this study.

### 2.6. Scanning Electron Microscopy (SEM)

**Figure 2:**
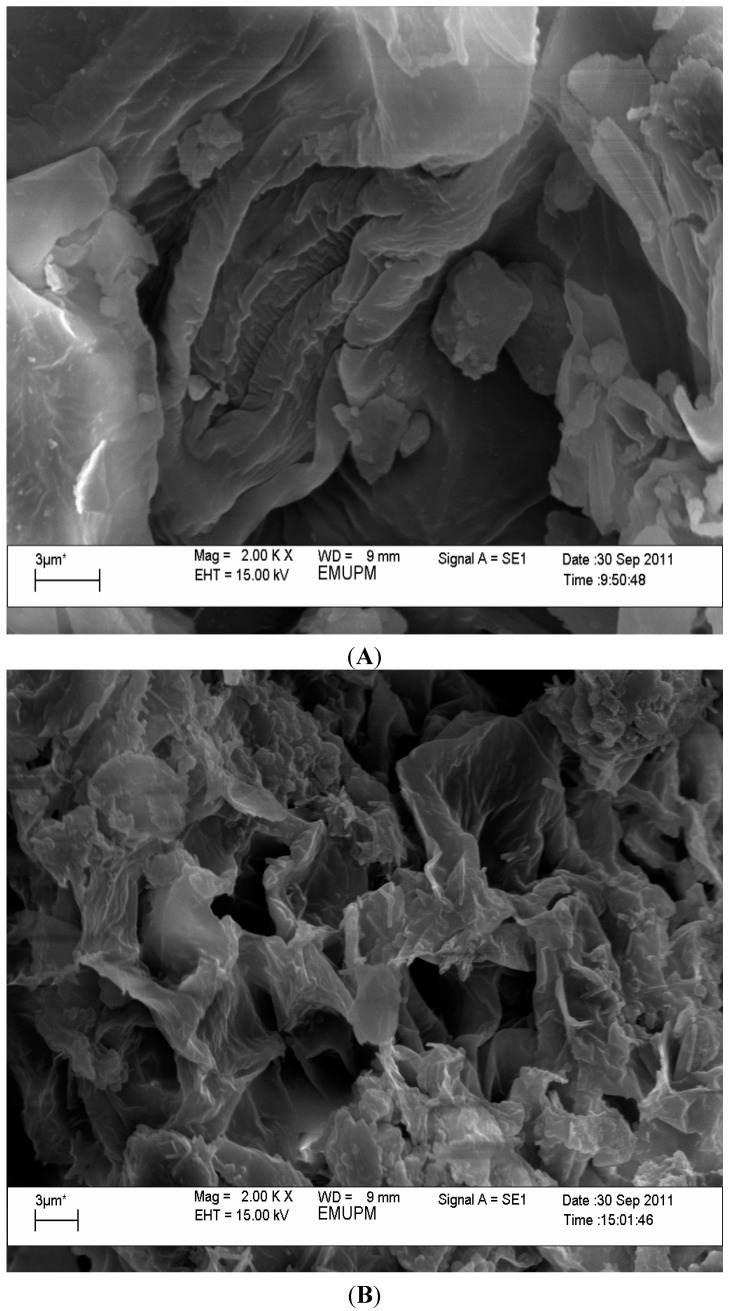
Scanning electron micrographs of (**A**) *Orthosiphon stamineus* before sonication treatment; (**B**) *Orthosiphon stamineus* after sonication treatment conditions at ethanol concentration: 54.13%, amplitude: 20%, duty cycle: 0.9 W/s and extraction time: 10 min.

Scanning electron micrographs of the *Orthosiphon stamineus* without and with sonication treatment are shown in Figure 2 to illustrate the cell damage caused by sonication treatment. UAE is able to increase the swelling of vegetal tissues, which causes pore enlargement in the plant cell wall, helps in diffusion and enhances the mass transfer. If the swelling vegetal tissue eventually breaks, it will facilitate the wash-out of the cell contents [23]. The surfaces of *Orthosiphon stamineus* without and with sonication treatment showed significant variations in shape and size when viewed by SEM. *Orthosiphon stamineus* before sonication showed an intact and smooth surface. In contrast, slight ruptures and porous walls were observed after sonication. This result could have occurred because the thin cell walls were not able to withstand the stresses of the high localized pressures and high temperatures applied by the UAE-generated cavitation bubbles. However, these changes in the plant matrix might allow for the easy entry of the solvent into the cellular channels [24]. Thus, this result suggests that UAE affects the internal structure of the plant cell, which enhances the efficiency of UAE. Several authors have suggested this mechanism for the extraction of caraway seeds [25] and rosemary leaves [26].

## 3. Experimental Section

### 3.1. Plant Material

One kilogram of *Orthosiphon stamineus* (Misai Kucing) in 200-mesh powder form was purchased from a local supplier (Ethno Resources Sdn Bhd) located in Selangor, Malaysia.

### 3.2. Chemicals and Reagents

All of the chemicals and solvents were of analytical reagent (AR) grade. The compounds 2,2'-azino-bis(3-ethylbenzothiazoline-6-sulfonic acid) diammonium salt (ABTS, ≈98% purity), 2,2'-diphenyl-1-picrylhydrazyl (DPPH, 95% purity), potassium persulfate (≥98% purity), sodium nitrite and (+)-catechin hydrate (≥98% purity) were purchased from Sigma-Aldrich (Steinheim, Germany). Folin-Ciocalteu’s phenol reagent, sodium carbonate (≥99.9% purity) and a sodium hydroxide solution (1 mol/L, 0.5 M) were purchased from Merck (Darmstadt, Germany). Gallic acid (98% purity) and Trolox (6-hydroxy-2,5,7,8-tetramethylchroman-2-carboxylic acid, 97% purity) were purchased from Acros Organics (Morris Plains, NJ, USA). Absolute ethanol (≥99.4%, v/v), denatured absolute ethanol (absolute ethanol/methanol 19:1) and aluminum chloride-6-hydrate (>99% purity) were purchased from Fisher Scientific Co. (Leicestershire, UK). Deionized water purified with a MilliQ water purification system (Millipore, Bedford, MA, USA) was used to prepare stock solutions and throughout the study.

### 3.3. Ultrasound-Assisted Extraction (UAE) from Orthosiphon Stamineus

UAE was performed by a Labsonic P sonifier (24 kHz, 400 W) from Sartorius (Goettingen, Germany) equipped with a cylindrical titanium alloy probe (14 mm diameter) in which an amber Schott bottle with the sample was placed. Extractions were carried out at a certain ethanol concentration (20%–100%), amplitude (20%–100%), duty cycle (0.1 W/s–1 W/s) and extraction time (2 min–10 min) according to the experimental design. First, 20 g of dried, ground sample were weighed and placed into an amber Schott bottle (250 mL) containing up to 200 mL of extracting solvent (solvent-to-solid ratio of 10:1). After a complete extraction, the herbal extract was filtered through a sand core glass funnel using Whatman No. 1 filter paper, and the clear solution of crude extract was collected in a light-protected amber bottle (125 mL) for further analysis without storage. All of the extractions were carried out in triplicate.

### 3.4. Experimental Design

Response surface methodology (RSM) was carried out to develop a second-order polynomial model for TPC, TFC, ABTS^•^^+^ and DPPH• responses. The type of central composite design (CCD) used in this study was central composite face-centered (CCF) to optimize the extraction of phenolic compounds. CCF was applied because it is convenient in optimizing a process with three levels (−1, 0 and +1) for each variable. The four independent variables were ethanol concentration (*X*_1_, %), amplitude (*X*_2_, %), duty cycle (*X*_3_, W/s) and extraction time (*X*_4_, min); the dependent variables (response variables) were TPC, TFC, ABTS^•^^+^ and DPPH• scavenging capacity, denoted *Y*_1_ (mg GAE/100 g DW), *Y*_2_ (mg CE/100 g DW), *Y*_3_ (µmol TEAC/100 g DW) and *Y*_4_ (µmol TEAC/100 g DW), respectively. A total of thirty different combinations with twenty-four factorial points and six center points were generated according to CCF configurations. Six replicate runs at the center points of the design were performed to allow for the estimation of pure error.

### 3.5. Analysis for Total Phenolic Content

The total phenolic content (TPC) was determined spectrophotometrically with Folin-Ciocalteu’s reagent (FCR) using a slightly modified version of the method [27]. One milliliter of properly diluted crude extract was pipetted into aluminum foil-wrapped test tubes (15 mL), followed by 1 mL of 10-fold diluted Folin-Ciocalteu’s reagent (FCR). After 4 min, 800 μL of a 75 g/L sodium carbonate solution was pipetted into the aluminum foil-wrapped test tubes. The contents of the tubes were mixed thoroughly with a vortex mixer (Model LMS, Tokyo, Japan) for 10 s and allowed to stand in the dark for 120 min at room temperature. The absorbance of the deep blue color that developed was measured against a blank at 765 nm using a Uvi Light Spectrophotometer (Model Genesys 10, Thermo Electron Corporation, Madison, WI, USA). The blank reagent was prepared by replacing 1 mL of crude extract with an equal amount of deionized water. Measurements were carried out in triplicate, and calculations were based on a calibration curve obtained with gallic acid (y = 13.51x, *R^2^* = 0.9816). The TPC was expressed as mg gallic acid equivalents (GAE) per 100 g dry weight (DW) of plant material.

### 3.6. Analysis for Total Flavonoid Content

Total flavonoid content (TFC) was determined using a slightly modified version of the standard method [28]. First, the extract (0.25 mL) was mixed with deionized water (1.25 mL) and a 5% (w/v) sodium nitrite solution (75 µL) in aluminum foil-wrapped test tubes (15 mL). After 6 min, a 10% (w/v) aluminum chloride solution (150 µL) was added, and the mixture was allowed to stand for 5 min before 1 M sodium hydroxide (0.5 mL) was added. Then, 275 µL of deionized water was transferred into the mixture, and it was mixed well for 10 s by a vortex mixer (Model LMS). The absorbance of the mixture was measured immediately at 510 nm. Measurements were carried out in triplicate, and calculations were based on a calibration curve obtained from (+)-catechin (y = 0.1685x − 0.0508, *R^2^* = 0.9647). The TFC were expressed as mg catechin equivalents (CE) per 100 g dry weight (DW) of herb sample. All analyses were performed in triplicate.

### 3.7. Determination of Antioxidant Activity—ABTS^•+^ Radical Cation

Antioxidant capacity was determined using the ABTS^•^^+^ radical cation decolorization assay according to a slightly modified method [29]. ABTS^•^^+^ radical cations were prepared by reacting 7 mM ABTS^•^^+^ with 2.45 mM potassium persulfate followed by incubation at room temperature in the dark for 16 h. The radicals are stable in this form for more than 2 days if stored in the dark at room temperature. The ABTS^•^^+^ solution then was diluted to an absorbance of 0.700 ± 0.02 at 734 nm using 95% denatured ethanol. The ABTS^•^^+^ solution (3.9 mL ABTS^•^^+^ solution, absorbance of 0.700 ± 0.02) was added to 0.1 mL of the crude extract and mixed thoroughly for 10 s with a vortex mixer. The reaction mixture was then allowed to stand at 25 °C for 6 min, and the absorbance at 734 nm was recorded immediately. A standard curve, y = 0.0459x + 9.52 (*R^2^* = 0.9839), was obtained with a Trolox [(±)6-hydroxy-2,5,7,8-tetramethylchromane-2-carboxylic acid] standard solution. The absorbances of the extracts were compared with that of the Trolox standard, and the results were expressed in terms of Trolox equivalent antioxidant capacity (TEAC) as micromoles of TEAC per 100 g dry weight (DW) of herb sample. All analyses were performed in triplicate.

### 3.8. Determination of Antioxidant Activity—DPPH• Radical Scavenging Capacity

The hydrogen atom or electron donation abilities were measured using the bleaching of a purple-colored ethanol solution of 2,2-diphenylpicrylhydrazyl (DPPH•) method with slight modifications [30]. Pure crude extract (0.1 mL) was transferred into an aluminum foil-wrapped test tube (15 mL), followed by ethanolic 0.1 mM DPPH• solution (3.9 mL). The DPPH• solution was vortexed using a vortex mixer (Model HYQ 3110, Thermo Line, Suzhou, China) for 1 min and then allowed to stand for 30 min in the dark before the absorbance was measured against a blank at 517 nm by a Uvi Light Spectrophotometer (Model Genesys 10, Thermo Electron Corporation, Madison, WI, USA). Spectrophotometric measurements were made using 3 mL of absolute ethanol as a blank, whereas deionized water was used to replace the 0.1 mL of pure crude extract in a negative control. The DPPH• scavenging capacity of the phenolic compounds was calculated as the percentage of DPPH• scavenging capacity using the following equation:

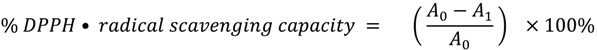
(5)
where A_0_ was the *A*_517_ of the control (containing deionized water and an ethanolic DPPH solution), and A_1_ was the *A*_517_ in the presence of the plant extract in an ethanolic DPPH solution.

The results were calculated using the Trolox [(±)6-hydroxy-2,5,7,8-tetramethylchromane-2-carboxylic acid] standard curve. The equation of the standard curve was y = 0.0364x + 7.0005 (*R*^2^ = 0.9904). The results were expressed in terms of Trolox equivalents antioxidant capacity (TEAC) as µmol TEAC per 100 g dry weight (DW) of plant material. All analyses were performed in triplicate.

### 3.9. Identification of Phenolics in Orthosiphon Stamineus Extract by HPLC-MS

High performance liquid chromatography-MS analysis was performed using a Thermo Finnigan model LCQ^DECA^ (San Jose, CA, USA) ion-trap mass spectrometer equipped with an ESI source connected to a surveyor HPLC binary pump, diode array detector (DAD) and auto sampler operated by Excalibur software (version 2.0, Thermo Scientific, Waltman, MA, USA). The mass spectra were collected under both negative and positive ion modes in the range of *m/z* 200–1,000. Chromatographic conditions were as follows: a Hypersil Gold analytical column (150 × 2.1 mm, 3 µm, Thermo Scientific) was used; the mobile phase was composed of solvent A, acetonitrile with 0.1% formic acid, and solvent B, deionized water with 0.1% formic acid; the injection volume was 20 µL; the flow rate was 250 µL/min; and the capillary temperature was 250 °C. A gradient program was performed as follows: the first 14 min were operated with the ratio of 10% A to 90% B, 15 min–24 min at a ratio of 35% A to 65% B, 25 min–39 min at a ratio of 70% A to 30% B and 40 min–50 min at a ratio of 100% A to 0% B.

### 3.10. Scanning Electron Microscopy (SEM)

*Orthosiphon stamineus* was mounted onto stubs, coated with gold and observed on a scanning electron microscope (LEO 1455 VPSEM with Oxford Inca EDX, Jena, Germany).

### 3.11. Statistical Analysis

Design-Expert (Version 7.1.4, Stat-Ease Inc., Minneapolis, MN, USA) was applied to design CCF and to analyze the experimental data in RSM. On the basis of the experimental data, regression coefficients were generated and were proposed as an empiric second-order polynomial model as shown in the following equation:

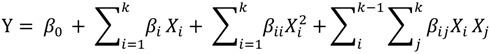
(6)
where *Y* is the response variable, *X_i_* and *X_j_* are the independent variables and *β_0_*, *β_i_*, *β_ii_*, and *β_ij_* are the regression coefficients for intercept, linear, quadratic and interaction terms, respectively. The significance of all terms in the polynomial was analyzed by computing the *F*-value at a probability (*p*) of 0.001, 0.01 or 0.05.

### 3.12. Verification of the Model

Optimal conditions for the extraction of phenolic compounds and the antioxidant capacity of the herbal extracts were obtained using the RSM second-order polynomial model, taking into consideration ethanol concentration, amplitude, duty cycle and extraction time. To find the points that maximized the responses, the numerical optimization method was adopted. A series of solutions was generated, and the solution to be used for the verification was selected on the basis of the highest desirability value and suitability. The experimental and predicted values of TPC, TFC, ABTS^•^^+^ and DPPH• scavenging capacity were compared to determine the validity of the model. To ensure the adequacy of the model, the whole extraction experiment was carried out in duplicate under selected optimized conditions.

## 4. Conclusions

The use of UAE was found to have a significant effect on the extraction efficiency of phenolic compounds obtained from an *Orthosiphon stamineus* extract, reported here for the first time. On the basis of the experimental results, it has been shown that different dependent responses favor different ethanol concentrations, amplitudes, duty cycles and extraction times. As for duty cycle, operation in pulse mode may be useful and more favorable than continuous mode to reduce the electrical energy consumption of the whole extraction process. In addition, ethanol was used as the extraction solvent as it is preferred as a solvent in the food industry and is regarded as a dietary alcohol. This study also indicated that *Orthosiphon stamineus* is a good source of essential functional components. The extraction variables, particularly ethanol concentration, significantly influenced the UAE of total phenolics, total flavonoids and antioxidants from *Orthosiphon stamineus*. Hence, UAE is well known as an ‘environmentally friendly’ or ‘green’ technique and may have strong potential in the near future as an efficient process for the preparation of extracts rich in natural antioxidants amidst growing environmental concerns.
